# Eribulin Mesylate Targets Human Telomerase Reverse Transcriptase in Ovarian Cancer Cells

**DOI:** 10.1371/journal.pone.0112438

**Published:** 2014-11-06

**Authors:** Satoko Yamaguchi, Yoshiko Maida, Mami Yasukawa, Tomoyasu Kato, Masayuki Yoshida, Kenkichi Masutomi

**Affiliations:** 1 Division of Cancer Stem Cell, National Cancer Center Research Institute, Tokyo, Japan; 2 Department of Gynecology, National Cancer Center Hospital, Tokyo, Japan; 3 Department of Pathology and Clinical Laboratories, National Cancer Center Hospital, Tokyo, Japan; Kanazawa University, Japan

## Abstract

Treatment of advanced ovarian cancer involves platinum-based chemotherapy. However, chemoresistance is a major obstacle. Cancer stem cells (CSCs) are thought to be one of the causes of chemoresistance, but the underlying mechanism remains elusive. Recently, human telomerase reverse transcriptase (hTERT) has been reported to promote CSC-like traits. In this study, we found that a mitotic inhibitor, eribulin mesylate (eribulin), effectively inhibited growth of platinum-resistant ovarian cancer cell lines. Eribulin-sensitive cells showed a higher efficiency for sphere formation, suggesting that these cells possess an enhanced CSC-like phenotype. Moreover, these cells expressed a higher level of hTERT, and suppression of hTERT expression by siRNA resulted in decreased sensitivity to eribulin, suggesting that hTERT may be a target for eribulin. Indeed, we found that eribulin directly inhibited RNA-dependent RNA polymerase (RdRP) activity, but not telomerase activity of hTERT *in* *vitro*. We propose that eribulin targets the RdRP activity of hTERT and may be an effective therapeutic option for CSCs. Furthermore, hTERT may be a useful biomarker to predict clinical responses to eribulin.

## Introduction

Ovarian cancer is the most lethal of all gynecological malignancies, claiming around 150,000 lives annually worldwide. The majority of ovarian cancers are diagnosed at an advanced stage, and platinum-based chemotherapy is the standard first-line treatment for advanced ovarian cancer patients. However, chemoresistance is a major obstacle in treating ovarian cancer.

Serous adenocarcinoma (SAC), the most common type of ovarian cancer, usually responds well to initial platinum-based chemotherapy, although it will recur and ultimately develop drug resistance. Clear cell carcinoma (CCC), the second most common type in Japan, is often resistant to initial platinum-based chemotherapy [1]. Regardless of whether the resistance is acquired or primary, more promising therapeutic strategies are necessary to overcome chemoresistance and improve the prognosis of ovarian cancer patients.

Recent studies have suggested that cancer stem cells (CSCs) are, at least in part, responsible for chemoresistance in many types of cancers including ovarian cancer [reviewed in [2]]. CSCs are a subpopulation of tumor cells, which are characterized by a self-renewal capacity and ability to differentiate into distinct cell types. The emergence of CSCs occurs at least partly as a result of epithelial-mesenchymal transition (EMT), a process essential for embryonic development, which is induced during cancer progression and crucial for cancer metastasis. CSCs possess the self-renewal feature of normal stem cells, and similar signaling pathways regulate self-renewal of CSCs and normal stem cells [3]. One such pathway involves telomerase reverse transcriptase (TERT), the rate-limiting catalytic subunit of telomerase, which is expressed in the majority of cancers. Recent evidence indicates that TERT regulates stem cell traits in a telomere length-independent manner. For example, TERT activates quiescent epidermal stem cells *in* *vivo* in a manner independent of the intrinsic RNA component of the telomerase enzyme TERC [4]. In addition, together with the SWItch-Sucrose NonFermentable (SWI-SNF) complex protein brahma-related gene 1 (BRG1), TERT acts as a transcriptional modulator of the Wnt/β-catenin signaling pathway, contributing to self-renewal and proliferation during development [5]. More recently, accumulating evidence indicates that TERT also operates in CSCs and promotes EMT and CSC-like traits. Specifically, overexpression of human TERT (hTERT) results in an enhanced sphere-forming capacity, increased expression of EMT/CSC markers, and increased *in* *vivo* tumorigenesis caused by hTERT interacting with β-catenin and enhancing its transcriptional activity [6]. Conversely, suppression of hTERT expression results in a decreased sphere-forming capacity and decreased expression of the CSC marker CD44 [7]. This function of hTERT in promotion of EMT and CSC-like traits appears to be independent of its telomerase activity [6]. Indeed, we have reported that hTERT in a complex with BRG1 and the nucleolar GTP-binding protein nucleostemin (NS) (TBN complex) participates in maintenance of CSCs. Moreover, we found that overexpression of the TBN complex enhances tumorigenicity and expression of EMT/CSC markers in an hTERT-dependent manner but in a telomere length-independent manner [8]. The exact telomerase-independent mechanisms by which the TBN complex regulates CSCs remain elusive. One possible mechanism is via the RNA-dependent RNA polymerase (RdRP) activity of hTERT [9]. RdRP induces RNA interference through production of double-stranded RNAs from single-stranded template RNAs and regulates the assembly of heterochromatin and mitotic progression [10]. Similar to RdRPs in model organisms, we found that the RdRP activities of the TBN complex are high in mitotic cells, and suppression of the TBN complex results in mitotic arrest [11].

To address chemoresistance, therapeutic strategies targeting EMT and CSCs are increasingly attracting attention. Recently, because eribulin mesylate (eribulin) was reported to inhibit metastasis by reversing EMT [12], we speculated that eribulin might target CSCs. Eribulin is a non-taxane inhibitor of microtubule dynamics [13], which induces irreversible mitotic blockade, leading to persistent inactivation of Bcl-2 and subsequent apoptosis [14]. In the United States, eribulin has been approved for treatment of metastatic breast cancer after at least two treatment regimens including an anthracycline and a taxane. Furthermore, eribulin is approved for treatment of inoperable or recurrent breast cancer in Japan.

In this study, we found that eribulin effectively inhibited growth of platinum-resistant ovarian cancer cells. Eribulin-sensitive cells showed enhanced CSC-like characteristics and high hTERT expression. Suppression of hTERT expression resulted in decreased sensitivity to eribulin. Moreover, eribulin inhibited the RdRP activity of hTERT *in* *vitro*, demonstrating that hTERT is a direct target of eribulin.

## Results

### Eribulin inhibits growth of cisplatin-resistant ovarian adenocarcinoma cell lines

**Figure 1:**
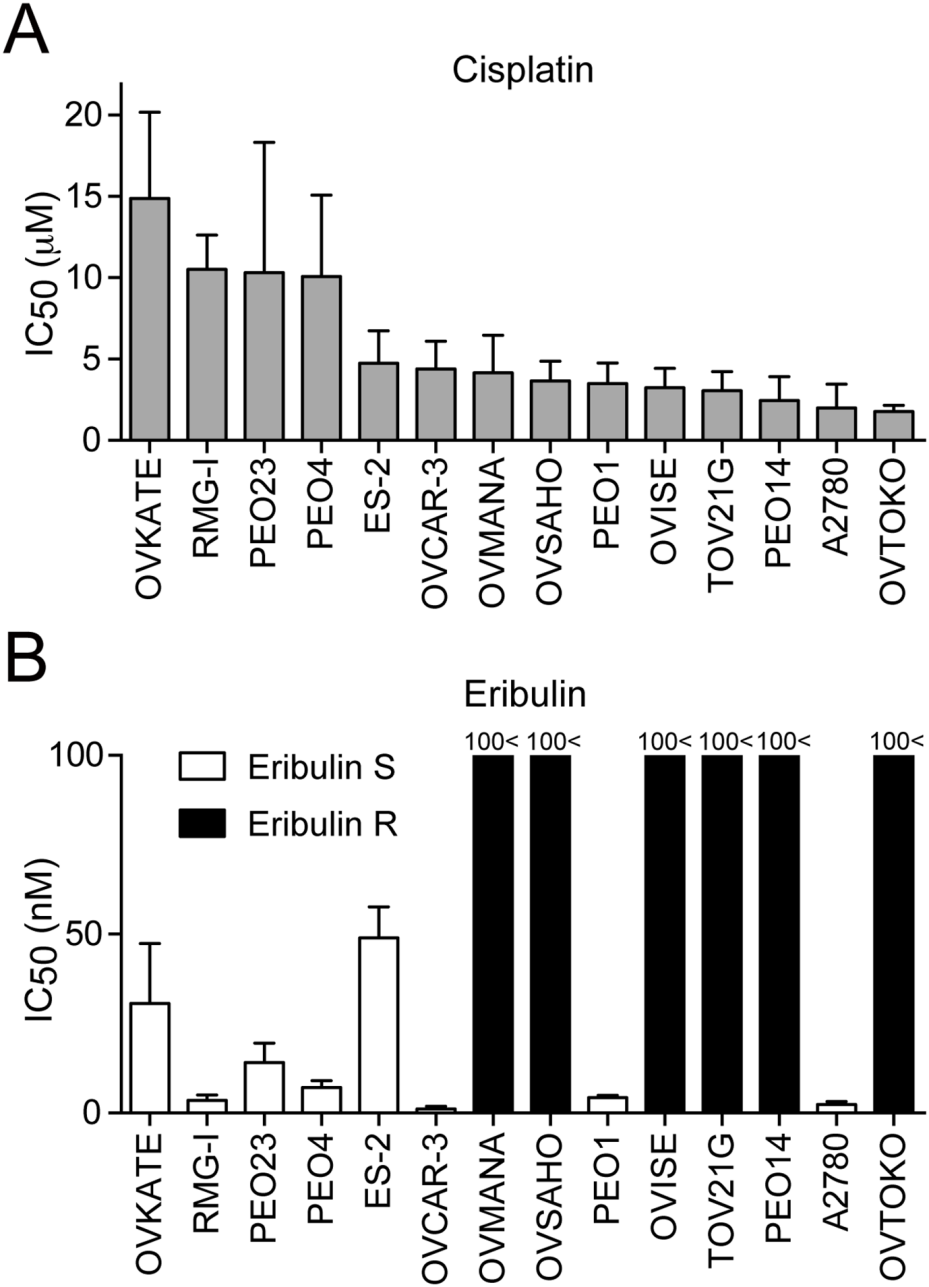
Eribulin inhibits growth of cisplatin-resistant ovarian cancer cells. Cells were treated with cisplatin or eribulin for 72 h, and then cell viability was determined by MTT assays. (A) Mean IC_50_ values for cisplatin (µM). (B) Mean IC_50_ values for eribulin (nM). Eribulin-sensitive (Eribulin S) cell lines are shown as open bars, and eribulin-resistant (Eribulin R) cell lines are shown as closed bars. Error bars represent the SD of at least three independent experiments.

Fourteen ovarian adenocarcinoma cell lines were investigated for sensitivity to cisplatin [cis-diamminedichloroplatinum(II)], including six SAC cell lines (PEO1, PEO4, PEO14, PEO23, OVKATE, and OVSAHO), six CCC cell lines (RMG-I, ES-2, OVISE, OVMANA, OVTOKO, and TOV21G), and two undifferentiated/unclassified adenocarcinoma cell lines (OVCAR-3 and A2780) (Table S1). As shown in Figure 1A, OVKATE, RMG-I, PEO4, and PEO23 cells were particularly resistant to cisplatin, presumably via different mechanisms. OVKATE cells have been previously reported as resistant to platinum agents with elevated expression of glutathione-S-transferase, a drug-resistance marker [15]. RMG-I cells are also resistant to cisplatin, which involves the extracellular signal-regulated kinase (ERK) pathway [16]. PEO4 and PEO23 cells were derived from the same patients as PEO1 and PEO14 cells, respectively, after development of clinical chemoresistance, and are therefore platinum resistant [17]. BRCA2 mutation has been found to contribute to platinum resistance in PEO4 cells [18].

We screened a series of known anti-cancer compounds for growth inhibition of platinum-resistant ovarian cancer cell lines. We found that eribulin, a mitotic inhibitor that suppresses microtubule dynamics [13], inhibited growth of RMG-I, PEO23, and PEO4 cells (Figure 1B). Strikingly, eribulin was not as effective in some of the cisplatin-sensitive cell lines such as OVTOKO, PEO14, and TOV21G (Figure 1A and 1B). For further characterization, we defined eight cell lines with an IC_50_ of <100 nM for eribulin as “eribulin-sensitive” (Eribulin S) and six cell lines with an IC_50_ of >100 nM for eribulin as “eribulin-resistant” (Eribulin R).

### Eribulin-sensitive cell lines show a higher sphere-forming capacity

**Figure 2:**
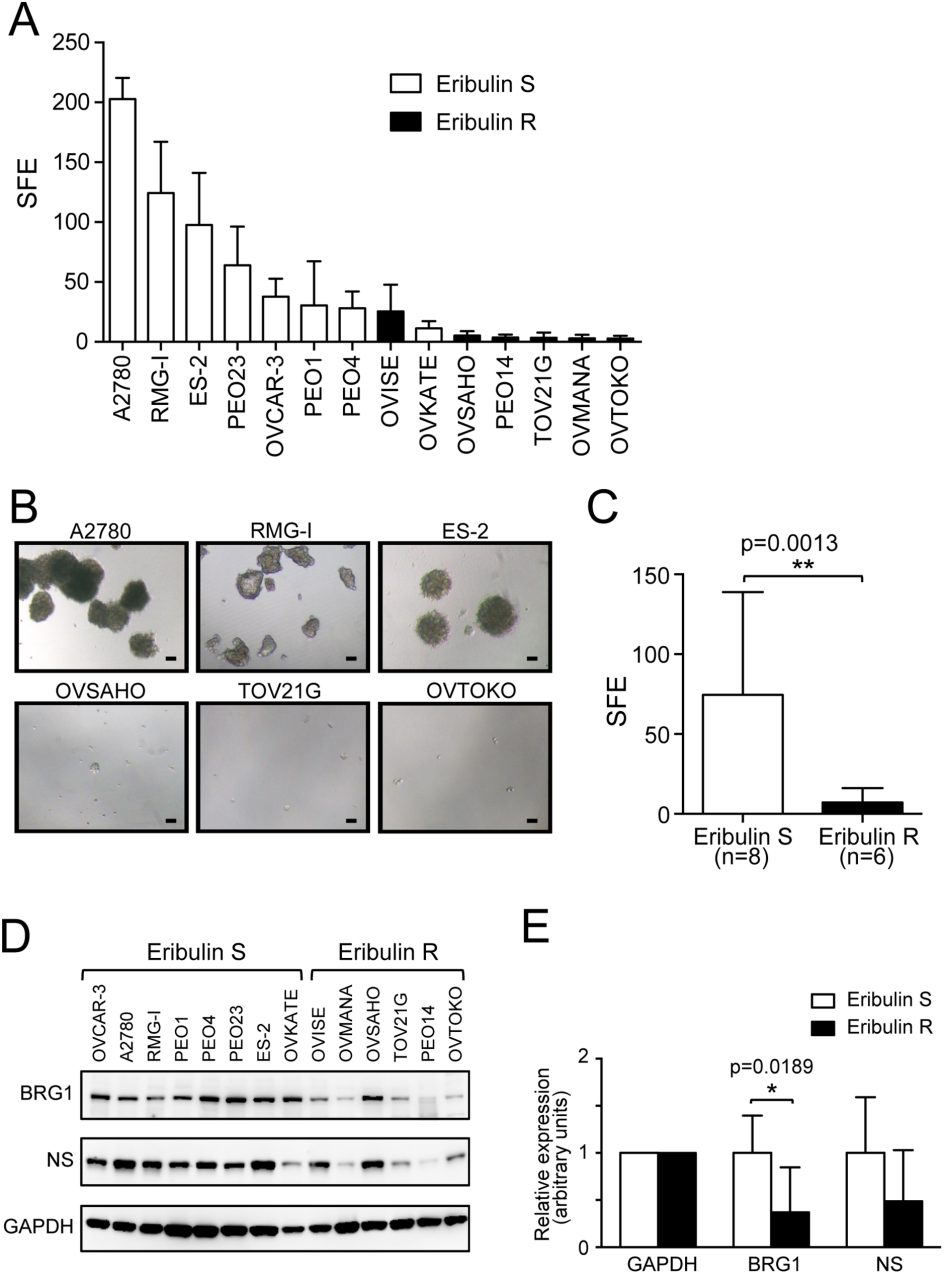
Eribulin-sensitive ovarian cancer cells show high sphere formation efficiency and higher BRG1 expression. (A) Sphere formation efficiency (SFE) of each cell line was indicated per 1,000 cells. Eribulin S cell lines are shown as open bars, and Eribulin R cells are shown as closed bars. Each experiment was performed at least three times, and mean values ± SD are indicated. (B) Morphology of tumorspheres under serum-free conditions. Representative images of spheres formed by A2780, RMG-I, ES-2, OVSAHO, TOV21G, and OVTOKO cells are shown. Scale bar = 50 µm. (C) The mean SFE of Eribulin S cell lines (n = 8) and Eribulin R cell lines (n = 6) shown in (A). Error bars indicate SD. (D) The level of BRG1 and NS protein expression was detected by immunoblotting. GAPDH expression was shown as loading control. (E) Signals in (D) were quantified with ImageJ software and normalized to GAPDH signal. The mean values of relative expression level ± SD are indicated.

CSCs are thought to be responsible for chemoresistance, and CSCs have been reported to contribute to cisplatin resistance in several types of cancer [19]. Moreover, it was recently reported that eribulin reverses EMT [12], a phenotype that is highly related to CSCs. Therefore, we investigated whether eribulin-sensitive cells possess an enhanced CSC-like phenotype. Because a sphere-forming capacity is a CSC-like characteristic, we performed sphere formation assays under serum-free conditions, and found that eribulin-sensitive cell lines showed high sphere formation efficiency (Figure 2A and 2B). The sphere formation efficiency of Eribulin S cell lines was significantly higher than that of Eribulin R cell lines (Figure 2C, p = 0.0013), suggesting that eribulin-sensitive cell lines possess enhanced CSC-like characteristics.

Since we have recently demonstrated that NS together with hTERT and BRG1 maintains CSCs [8] and we and others have also demonstrated that NS is a useful CSC marker [20]–[22], we investigated the expression of BRG1 and NS in Eribulin S and Eribulin R cell lines. The protein expression level of BRG1 was significantly higher in Eribulin S cell lines (Figure 2D and 2E, p = 0.0189), while only a modest tendency of higher level of NS in Eribulin S cell lines was observed (Figure 2D and 2E, p = 0.1216). We did not detect a difference in the expression level of CD133 or CD44 (Figure S1), the cell surface markers implied in some CSCs [23].

### Eribulin S cells express higher levels of hTERT protein

**Figure 3:**
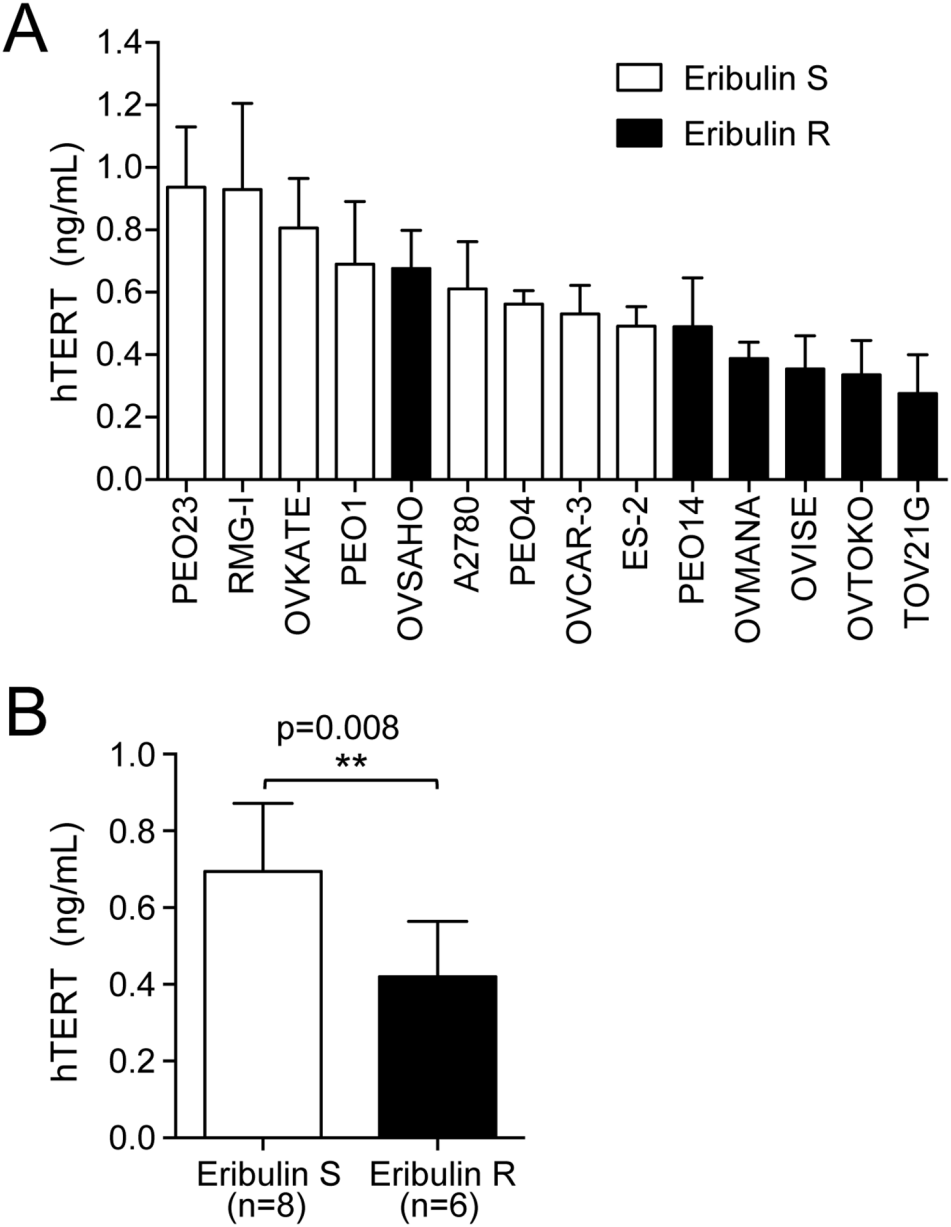
Eribulin-sensitive ovarian cancer cells express higher levels of hTERT protein. (A) The level of hTERT protein expression was determined by ELISA (indicated as ng/ml). Eribulin S cell lines are shown as open bars, and Eribulin R cells are shown as closed bars. Each experiment was performed at least three times, and mean values ± SD are indicated. (B) The mean hTERT level of Eribulin S cell lines (n = 8) and Eribulin R cell lines (n = 6) shown in (A). Error bars indicate SD.

Overexpression of hTERT results in an enhanced sphere-forming capacity in gastric cancer cells [6]. Conversely, suppression of hTERT expression results in a decreased sphere-forming capacity in breast cancer cells [7]. Therefore, we determined whether the ovarian cancer cells with a higher sphere-forming capacity express a higher level of hTERT. We observed a tendency in cell lines with high sphere-forming efficiency, such as RMG-I, PEO23, and A2780, to express relatively high levels of hTERT protein, while cell lines with low sphere-forming efficiency, such as TOV21G, OVTOKO, and OVMANA, expressed low levels of hTERT protein, as demonstrated by enzyme-linked immunosorbent assay (ELISA) (Figure 3A). The high level of hTERT expression in RMG-I cells can be accounted for by a gain-of-function mutation (-124 G>A) in the hTERT promoter region (Table S1 and Figure S2). This cancer-specific mutation was recently reported in melanoma and several other types of cancer [24]–[27], which creates new binding motifs for E-twenty six/ternary complex factors (ETS/TCF) and thus contributes to upregulated hTERT transcription [24], [25].

We found that Eribulin S cell lines expressed higher levels of hTERT protein than those in Eribulin R cell lines (Figure 3B, p = 0.008).

### Suppression of hTERT expression results in decreased sensitivity to eribulin

**Figure 4:**
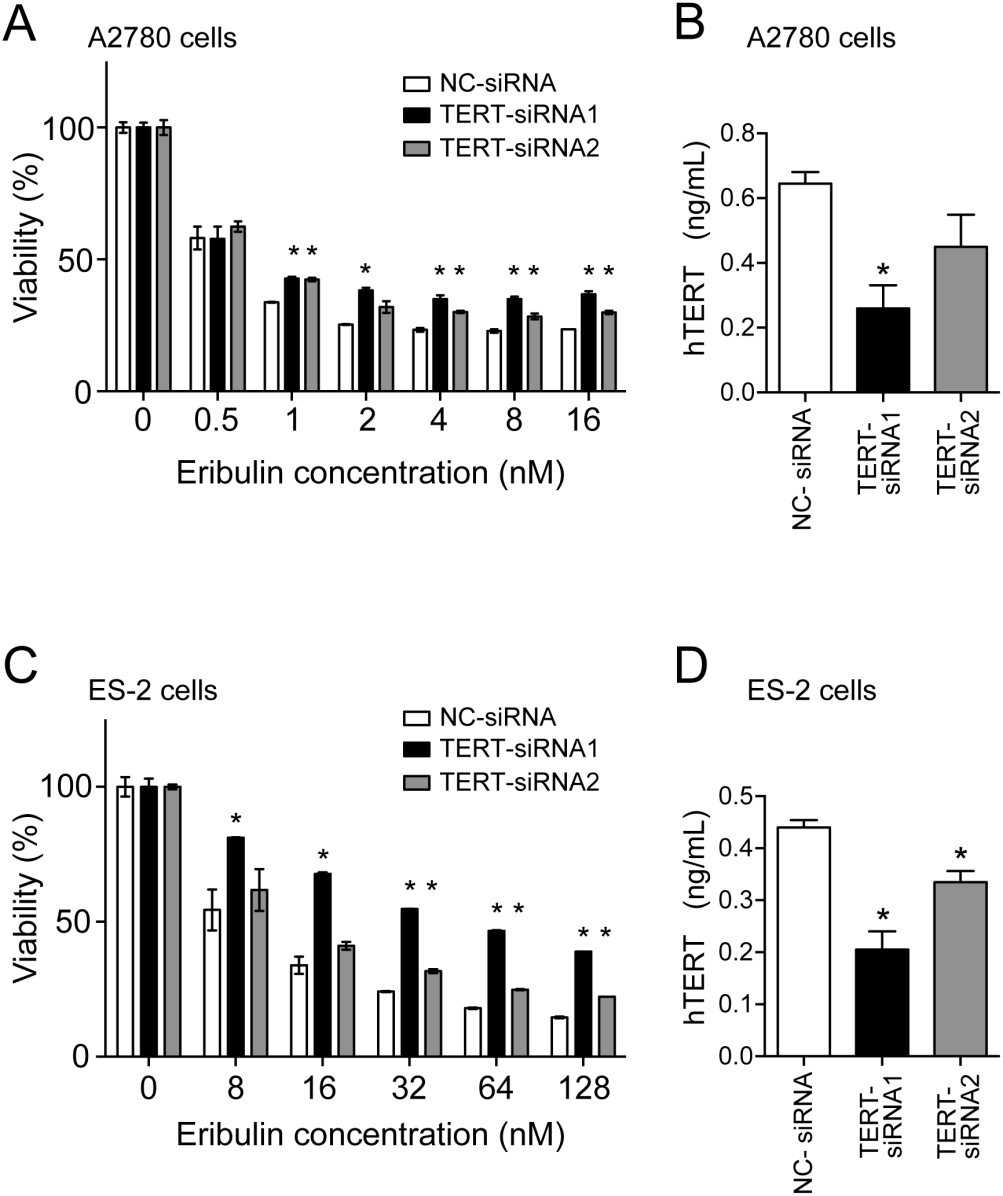
Suppression of hTERT expression by siRNA results in decreased sensitivity to eribulin. (A) A2780 cells expressing control siRNA (open bars), TERT siRNA1 (closed bars), or TERT siRNA2 (shaded bars) were treated with eribulin for 72 h, and then cell viability was determined by MTT assays. *p<0.05 vs. cells expressing control siRNA. (B) The level of hTERT protein expression in A2780 cells expressing control siRNA (open bars), TERT siRNA1 (closed bars), or TERT siRNA2 (shaded bars) was determined by ELISA (indicated as ng/ml). (C and D) The experiments described in (A and B) were performed in the same manner using ES-2 cells expressing control siRNA (open bars), TERT siRNA1 (closed bars), or TERT siRNA2 (shaded bars). *p<0.05 vs. cells expressing control siRNA.

The correlation between hTERT expression and eribulin sensitivity led us to postulate that eribulin inhibits growth of ovarian cancer cells via inhibition of hTERT. To test this hypothesis, we examined whether suppression of hTERT expression in ovarian cancer cells leads to decreased sensitivity to eribulin. Two independent hTERT-specific siRNAs were introduced into A2780 cells, and sensitivity to eribulin was compared with cells expressing control siRNA. As expected, cells expressing hTERT siRNAs showed decreased sensitivity to eribulin (Figure 4A). TERT siRNA1 showed a tendency of stronger suppression of hTERT expression than TERT siRNA2 as demonstrated by ELISA (Figure 4B). This finding may explain why cells expressing TERT siRNA1 tended to be less sensitive to eribulin than those expressing TERT siRNA2 (Figure 4A). Similar results were obtained in ES-2 cells (Figure 4C and 4D). These results suggest that hTERT might be a direct target for eribulin.

### Eribulin inhibits RdRP activity of hTERT *in* *vitro*

**Figure 5:**
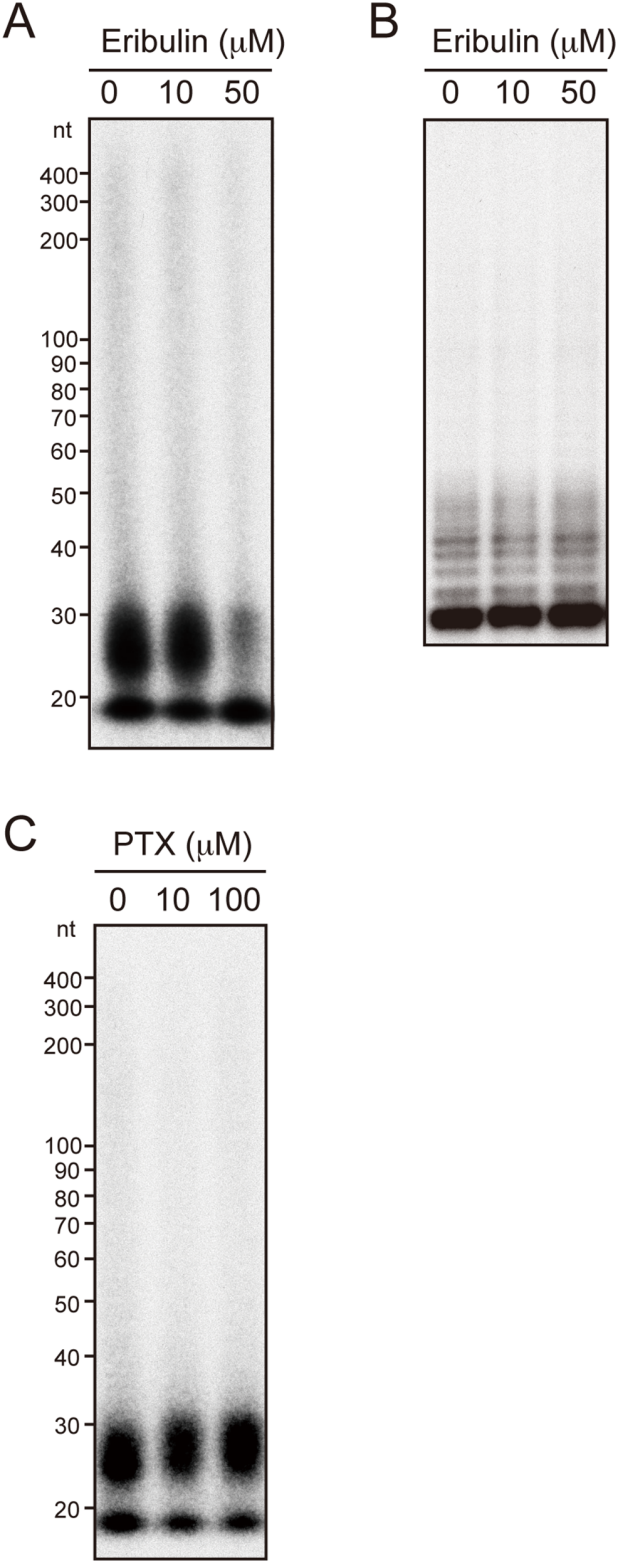
Eribulin inhibits RdRP activity but not telomerase activity of hTERT. (A) RdRP activity of hTERT immune complexes prepared from HeLa cells arrested in the mitotic phase was assayed without or with 10 and 50 µM eribulin. (B) Telomerase activity in HeLa cell extracts was assayed without or with 10 and 50 µM eribulin. (C) RdRP activity of hTERT immune complexes was assayed without or with 10 and 100 µM paclitaxel (PTX).

It is widely believed that any effect of hTERT suppression is mediated by telomere shortening. However, because we observed decreased sensitivity to eribulin in a relatively short period (Figure 4, 96 h after transfection of siRNA against hTERT), we speculated that this effect is independent of the telomere maintenance function of hTERT. Moreover, the function of hTERT in promotion of EMT and CSC-like traits is independent of its telomerase activity [6]. Together with our recent report showing that hTERT has an RdRP activity independent of telomere maintenance [9], we investigated whether eribulin directly targets hTERT-RdRP activity. We monitored the inhibitory effect of eribulin on hTERT-RdRP activity using an *in* *vitro* RdRP assay [11], and found that eribulin inhibited hTERT-RdRP activity *in* *vitro* at a concentration of 50 µM (Figure 5A). The same concentration of eribulin did not inhibit the telomerase activity of hTERT as shown by telomeric repeat amplification protocol (TRAP) assay (Figure 5B). These results suggest that the effects of eribulin on hTERT are not mediated via telomerase activity, but via RdRP activity. Interestingly, another mitotic inhibitor, paclitaxel, a representative taxane, did not inhibit RdRP activity (Figure 5C), suggesting that eribulin has a specific inhibitory effect on hTERT-RdRP activity.

## Discussion

Among gynecological cancers, ovarian cancer is the leading cause of death. In particular, resistance to conventional platinum-based chemotherapy has been a barrier in the improvement of prognoses for ovarian cancer patients, and new therapeutic strategies are urgently required. Here, we found that eribulin was effective to inhibit growth of platinum-resistant ovarian cancer cells. Effects of eribulin were correlated with hTERT expression levels (Figure 3), and suppression of hTERT expression resulted in decreased sensitivity to eribulin (Figure 4), suggesting that hTERT could be a target of eribulin in these cells. Indeed, eribulin inhibited the RdRP activity but not the reverse transcriptase activity of hTERT *in* *vitro* (Figure 5).

### CSCs and hTERT

CSCs are thought to be involved in chemoresistance, and several pathways have been found to contribute to the promotion or maintenance of CSCs. We and others have shown that hTERT plays an important role in promotion and maintenance of CSCs in telomere maintenance-independent manners [6]–[8]. Eribulin effectively inhibited growth of platinum-resistant cells (Figure 1). Eribulin-sensitive cells exhibited higher hTERT expression (Figure 3) and a higher sphere-forming capacity (Figure 2), suggesting that these cells have enhanced CSC-like characteristics, possibly due to the high levels of hTERT protein. Consistently, eribulin-sensitive cells exhibited higher BRG1 expression (Figure 2), another component of the TBN complex that maintains CSCs. We did not detect a significant difference in the expression of CD133 or CD44 (Figure S1). Although CD133 and CD44 are thought to be indicative of CSCs in some types of cancer, it remains to be elucidated what markers are appropriate for CSCs in ovarian cancers [23].

Because telomere maintenance by telomerase is indispensable for infinite proliferation of malignant cells, efforts have been made to develop anticancer therapeutics targeting telomerase. Recent studies indicate that TERT plays functional roles beyond telomere maintenance. Indeed, the function of TERT to activate normal quiescent stem cells or CSC-like traits has been shown to be independent of its telomerase activity [4], [6], [28]. We have also found that the TBN complex maintains CSCs in a telomere length-independent manner [8]. It is possible that this telomerase-independent mechanism is mediated by the RdRP activity of TERT, because the TBN complex itself is responsible for the RdRP activity and is involved in heterochromatin regulation and mitotic progression [11]. We speculate that the RdRP activity of hTERT is involved in gene expression through heterochromatin regulation in cancer cells, and it could be a novel anticancer therapeutic target. Whether RdRP activity is prerequisite for hTERT function in the promotion of CSCs remains to be determined.

### Eribulin and hTERT

Eribulin binds to microtubule plus ends and inhibits the growth phase of microtubule dynamics [29]. Recently, eribulin was shown to reverse EMT by downregulating transforming growth factor-β (TGF-β)-induced Smad phosphorylation [12]. Smad proteins bind to microtubules in the absence of TGF-β and TGF-β triggers dissociation from microtubules and phosphorylation of Smad proteins [30]. Yoshida *et al.* speculated that eribulin inhibits Smad phosphorylation possibly by suppressing Smad dissociation from microtubules [12]. Because we have recently demonstrated that hTERT localizes to mitotic spindles and centromeres during mitosis [11], it is also possible that eribulin inhibits hTERT functions by interfering with the interaction between hTERT and microtubules. Eribulin improves overall survival of patients with metastatic breast cancer, who had prior anthracycline- and taxane-based chemotherapy [31]. A taxane drug, paclitaxel, did not inhibit the RdRP activity of hTERT (Figure 5C), providing one of the potential molecular bases for the different clinical outcomes of taxanes and eribulin. The exact mechanism by which eribulin inhibits hTERT function is yet to be understood.

### hTERT as a biomarker

It is important to identify biomarkers to predict responses to anticancer therapies. By determination of hTERT levels in clinical specimens, patients who are likely to respond well to eribulin may be identified before they receive chemotherapy. In particular, an ELISA would be able to measure hTERT levels in clinical practice.

In summary, we found that eribulin inhibits RdRP activity of hTERT, which may contribute to chemoresistance in ovarian cancer by maintaining CSCs. Eribulin inhibited the growth of ovarian cancer cells with high hTERT expression and strong platinum resistance, suggesting it may be a promising therapeutic agent for chemoresistant ovarian cancer. Moreover, hTERT may be a useful biomarker to predict clinical responses to eribulin.

## Materials and Methods

### Cell lines

RMG-I [32], OVMANA [33], OVTOKO [34], OVISE [34], OVSAHO [33], and OVKATE [33] cells were obtained from the Japanese Collection of Research Bioresources Cell Bank. OVCAR-3 cells [35] were obtained from the RIKEN BioResource Center. PEO1, PEO4, PEO14, PEO23 [17], and A2780 [36] cells were purchased from the European Collection of Cell Cultures, and TOV21G [37] and ES-2 [38] cells were purchased from the American Type Culture Collection. RMG-I cells were cultured in Ham’s F12 medium supplemented with 10% fetal bovine serum, ES-2 cells in McCoy’s 5a medium supplemented with 10% fetal bovine serum, TOV21G cells in MCDB105/Medium 199 (1∶1) supplemented with 10% fetal bovine serum, and HeLa cells in Dulbecco’s modified Eagle’s medium (DMEM) supplemented with 10% fetal bovine serum. All other cell lines (A2780, OVCAR-3, OVMANA, OVTOKO, OVISE, OVSAHO, OVKATE, PEO1, PEO4, PEO14, and PEO23) were cultured in RPMI-1640 medium supplemented with 10% fetal bovine serum and 1 mM sodium pyruvate (Gibco, Grand Island, NY, USA).

### Compounds

Cisplatin was purchased from Sigma-Aldrich (St Louis, MO, USA), paclitaxel was purchased from Wako (Osaka, Japan), and eribulin (Halaven) was purchased from Eisai Co., Ltd (Tsukuba, Japan).

### MTT assay

Cells (5,000–10,000 per well) were seeded in 96-well plates and then treated with cisplatin or eribulin after 24 h. At 72 h of treatment, an MTT proliferation assay (Cell Proliferation Kit I MTT, Roche Diagnostics, Mannheim, Germany) was performed according to the manufacturer’s protocol. Briefly, 10 µl MTT labeling reagent was added to each well, followed by 4 h of incubation. Then, 100 µl solubilization solution was added to each well, followed by overnight incubation. The reaction product was quantified by measuring the absorbance at 570 and 690 nm using a microplate reader (Viento 808, BioTek, Winooski, VT, USA). Cell viability was determined by comparisons to untreated cells.

### Sphere formation assay

Single cells were seeded in 96-well ultra low attachment plates (Corning Inc, Corning, NY, USA) at 100–1,000 cells/100 µl medium in each well. Cells were grown in serum-free DMEM/F12 medium (Gibco) supplemented with 20 ng/ml basic fibroblast growth factor (Wako), 20 ng/ml epidermal growth factor (Wako), and B27 supplement (Gibco). Cultures were supplemented with 25 µl of fresh medium every 3–4 days, and the number of spheres was counted on days 7 and 14. Microscopic images were obtained with a CKX41 inverted microscope and DP21 digital camera (Olympus, Tokyo, Japan).

### Immunoblotting

Cells were lysed in radioimmunoprecipitation assay (RIPA) buffer containing 1% NP-40, 1 mM EDTA, 50 mM Tris-HCl (pH 7.4) and 150 mM NaCl. After sonication and centrifugation of the lysates, proteins (20 µg) were subjected to SDS-PAGE in 7.5% poly-acrylamide gels, followed by immunoblot analysis. The following antibodies were used: anti-BRG1 (a gift from Dr. Tsutomu Ohta, National Cancer Center, Japan), anti-NS (A300–600A; Bethyl Laboratories, Montgomery, TX, USA), anti-GAPDH (3H12; Medical & Biological Laboratories (MBL), Nagoya, Japan), anti-CD133 (W6B3C1; Miltenyi Biotec, Bergisch Gladbach, Germany) and anti-CD44 (2C5; R&D Systems, Minneapolis, MN, USA). Signals were detected by LAS-3000 (Fujifilm, Tokyo, Japan), quantified with ImageJ software (National Institutes of Health, USA) and normalized using GAPDH loading control.

### hTERT ELISA

The hTERT ELISA employed a rabbit anti-hTERT polyclonal antibody as the capture antibody (MBL), and a mouse anti-hTERT monoclonal antibody (mAb) (clone 2E4-5) as the detection antibody (MBL code no. 5340, Ab-Match Assembly Human TERT Kit). The 2E4-5 antibody was generated against recombinant hTERT as an immunogen as described previously [11]. Cells were lysed in RIPA buffer. After sonication and centrifugation of the lysates, 100 µg total protein (100 µl in volume) was added to each well of a 96-well plate (MBL code no. 5310, Ab-Match Universal Kit). The ELISA was performed according to the manufacturer’s instructions. Absorbances at 450 and 630 nm were measured by a microplate reader. Each experiment was performed at least three times, and mean values were calculated.

### TERT promoter mutation analysis

Genomic DNA was extracted from ovarian cancer cell lines using a Blood and Cell Culture DNA Kit (Qiagen, Hilden, Germany) according to the manufacturer’s protocol. The *TERT* promoter region (−146 to −124-bp upstream from the start codon) was amplified by PCR using KOD FX (Toyobo, Osaka, Japan) and the following primers: 5′-GTCCTGCCCCTTCACCTT-3′ and 5′-CAGCGCTGCCTGAAACTC-3′ [25]. PCR was performed under the following conditions: 40 cycles of 98°C for 10 s, 55°C for 30 s, and 68°C for 60 s. Purified PCR products were sequenced by Sanger sequencing.

### Transfection of siRNA

Cells were transfected with siRNA by Lipofectamine RNAiMAX (Invitrogen) and then seeded at 5,000–10,000 cells per well in 96-well plates. At 24 h after transfection, the cells were treated with eribulin, and an MTT assay was performed after 72 h of treatment. For the ELISA, 2–5.0×10^6^ cells transfected with siRNA were plated in a 10-cm petri dish, and then collected after 48 h of incubation. hTERT siRNA1 and hTERT siRNA2 have been described previously [8]. The negative control siRNA (MISSION siRNA Universal Negative Control; Sigma-Aldrich) was also used.

### IP-RdRP assay

In order to detect RdRP activity *in* *vitro*, the hTERT immune complex was isolated by mAb against hTERT. An IP-RdRP assay has been established in mitotically arrested HeLa cells [11]. Therefore, HeLa cells were used for this assay. To synchronize HeLa cells undergoing mitosis, the cells were cultured in medium containing 2.5 mM thymidine (Nacalai Tesque, Kyoto, Japan) for 24 h. At 6 h after release, the cells were incubated in medium containing 0.1 µg/ml nocodazole (Sigma-Aldrich) for 14 h. After shaking gently, mitotic cells were retrieved. The IP-RdRP assay was performed as described previously [11].

### TRAP assay

A TRAP assay was used to detect telomere specific reverse transcriptase activity as described previously [39].

### Statistical analysis

Statistical analyses were performed with GraphPad Prism 6 (GraphPad Software, La Jolla, CA, USA). The Student’s t-test or Mann Whitney test was used. Two-sided p-values of <0.05 were considered statistically significant.

## Supporting Information

Figure S1 **CD133 and CD44 expression in Eribulin S and Eribulin R ovarian cancer cells.** (A) The level of CD133 and CD44 protein expression was detected by immunoblotting. Since the data was obtained in the same experiment as Figure 2 panel D, GAPDH gel was identical with Figure 2 panel D. (B) Signals in (A) were quantified with ImageJ software and normalized to GAPDH signal. The mean values of relative expression level ± SD are indicated.
(TIF)

Figure S2 **ES-2 and RMG-I cells possess hTERT promoter mutations.** The hTERT promoter was sequenced in each cell line. ES-2 cells harbor a -138/-139 GG>AA mutation as described previously [27], and RMG-I cells harbor a -124 G>A mutation. The wild-type sequences of the corresponding regions from OVKATE and OVSAHO cells are shown as controls.
(TIF)

Table S1 **Ovarian cancer cell lines used in this study.**
(DOCX)

## References

[1] Takano M, Tsuda H, Sugiyama T (2012) Clear cell carcinoma of the ovary: is there a role of histology-specific treatment? J Exp Clin Cancer Res 31: 53. 22655678 10.1186/1756-9966-31-53 PMC3405444

[2] Singh A, Settleman J (2010) EMT, cancer stem cells and drug resistance: an emerging axis of evil in the war on cancer. Oncogene 29: 4741–4751. 20531305 10.1038/onc.2010.215 PMC3176718

[3] Pardal R, Clarke MF, Morrison SJ (2003) Applying the principles of stem-cell biology to cancer. Nat Rev Cancer 3: 895–902. 14737120 10.1038/nrc1232

[4] Sarin KY, Cheung P, Gilison D, Lee E, Tennen RI, et al (2005) Conditional telomerase induction causes proliferation of hair follicle stem cells. Nature 436: 1048–1052. 16107853 10.1038/nature03836 PMC1361120

[5] Park JI, Venteicher AS, Hong JY, Choi J, Jun S, et al (2009) Telomerase modulates Wnt signalling by association with target gene chromatin. Nature 460: 66–72. 19571879 10.1038/nature08137 PMC4349391

[6] Liu Z, Li Q, Li K, Chen L, Li W, et al (2013) Telomerase reverse transcriptase promotes epithelial-mesenchymal transition and stem cell-like traits in cancer cells. Oncogene 32: 4203–4213. 23045275 10.1038/onc.2012.441

[7] Chung SS, Aroh C, Vadgama JV (2013) Constitutive Activation of STAT3 Signaling Regulates hTERT and Promotes Stem Cell-Like Traits in Human Breast Cancer Cells. PLoS One 8: e83971. 24386318 10.1371/journal.pone.0083971 PMC3875492

[8] Okamoto N, Yasukawa M, Nguyen C, Kasim V, Maida Y, et al (2011) Maintenance of tumor initiating cells of defined genetic composition by nucleostemin. Proc Natl Acad Sci U S A 108: 20388–20393. 21730156 10.1073/pnas.1015171108 PMC3251068

[9] Maida Y, Yasukawa M, Furuuchi M, Lassmann T, Possemato R, et al (2009) An RNA-dependent RNA polymerase formed by TERT and the RMRP RNA. Nature 461: 230–235. 19701182 10.1038/nature08283 PMC2755635

[10] Martienssen RA, Zaratiegui M, Goto DB (2005) RNA interference and heterochromatin in the fission yeast Schizosaccharomyces pombe. Trends Genet 21: 450–456. 15979194 10.1016/j.tig.2005.06.005

[11] Maida Y, Yasukawa M, Okamoto N, Ohka S, Kinoshita K, et al (2014) Involvement of telomerase reverse transcriptase in heterochromatin maintenance. Mol Cell Biol 34: 1576–1593. 24550003 10.1128/MCB.00093-14 PMC3993606

[12] Yoshida T, Ozawa Y, Kimura T, Sato Y, Kuznetsov G, et al (2014) Eribulin mesilate suppresses experimental metastasis of breast cancer cells by reversing phenotype from epithelial-mesenchymal transition (EMT) to mesenchymal-epithelial transition (MET) states. Br J Cancer 110: 1497–1505. 24569463 10.1038/bjc.2014.80 PMC3960630

[13] Towle MJ, Salvato KA, Budrow J, Wels BF, Kuznetsov G, et al (2001) In vitro and in vivo anticancer activities of synthetic macrocyclic ketone analogues of halichondrin B. Cancer Res. 61: 1013–1021. 11221827

[14] Towle MJ, Salvato KA, Wels BF, Aalfs KK, Zheng W, et al (2011) Eribulin induces irreversible mitotic blockade: implications of cell-based pharmacodynamics for in vivo efficacy under intermittent dosing conditions. Cancer Res 71: 496–505. 21127197 10.1158/0008-5472.CAN-10-1874

[15] Ohta I, Gorai I, Miyamoto Y, Yang J, Zheng JH, et al (2001) Cyclophosphamide and 5-fluorouracil act synergistically in ovarian clear cell adenocarcinoma cells. Cancer Lett 162: 39–48. 11121861 10.1016/s0304-3835(00)00605-4

[16] Wang J, Zhou JY, Wu GS (2011) Bim protein degradation contributes to cisplatin resistance. J Biol Chem 286: 22384–22392. 21561860 10.1074/jbc.M111.239566 PMC3121386

[17] Langdon SP, Lawrie SS, Hay FG, Hawkes MM, McDonald A, et al (1988) Characterization and properties of nine human ovarian adenocarcinoma cell lines. Cancer Res 48: 6166–6172. 3167863

[18] Sakai W, Swisher EM, Jacquemont C, Chandramohan KV, Couch FJ, et al (2009) Functional restoration of BRCA2 protein by secondary BRCA2 mutations in BRCA2-mutated ovarian carcinoma. Cancer Res 69: 6381–6386. 19654294 10.1158/0008-5472.CAN-09-1178 PMC2754824

[19] Vidal SJ, Rodriguez-Bravo V, Galsky M, Cordon-Cardo C, Domingo-Domenech J (2014) Targeting cancer stem cells to suppress acquired chemotherapy resistance. Oncogene 33: 4451–4463. 24096485 10.1038/onc.2013.411

[20] Tamase A, Muraguchi T, Naka K, Tanaka S, Kinoshita M, et al (2009) Identification of tumor-initiating cells in a highly aggressive brain tumor using promoter activity of nucleostemin. Proc Natl Acad Sci U S A 106: 17163–17168. 19805150 10.1073/pnas.0905016106 PMC2761321

[21] Kobayashi T, Masutomi K, Tamura K, Moriya T, Yamasaki T, et al (2014) Nucleostemin expression in invasive breast cancer. BMC Cancer 14: 215. 24650343 10.1186/1471-2407-14-215 PMC3994431

[22] Lin T, Meng L, Li Y, Tsai RY (2010) Tumor-initiating function of nucleostemin-enriched mammary tumor cells. Cancer Res 70: 9444–9452. 21045149 10.1158/0008-5472.CAN-10-2159 PMC2982898

[23] Medema JP (2013) Cancer stem cells: the challenges ahead. Nat Cell Biol 15: 338–344. 23548926 10.1038/ncb2717

[24] Huang FW, Hodis E, Xu MJ, Kryukov GV, Chin L, et al (2013) Highly recurrent TERT promoter mutations in human melanoma. Science 339: 957–959. 23348506 10.1126/science.1229259 PMC4423787

[25] Horn S, Figl A, Rachakonda PS, Fischer C, Sucker A, et al (2013) TERT promoter mutations in familial and sporadic melanoma. Science 339: 959–961. 23348503 10.1126/science.1230062

[26] Killela PJ, Reitman ZJ, Jiao Y, Bettegowda C, Agrawal N, et al (2013) TERT promoter mutations occur frequently in gliomas and a subset of tumors derived from cells with low rates of self-renewal. Proc Natl Acad Sci U S A 110: 6021–6026. 23530248 10.1073/pnas.1303607110 PMC3625331

[27] Wu RC, Ayhan A, Maeda D, Kim KR, Clarke BA, et al (2013) Frequent somatic mutations of the telomerase reverse transcriptase promoter in ovarian clear cell carcinoma but not in other major types of gynecologic malignancies. J Pathol 232: 473–481. 10.1002/path.4315 PMC3946218 24338723

[28] Choi J, Southworth LK, Sarin KY, Venteicher AS, Ma W, et al (2008) TERT promotes epithelial proliferation through transcriptional control of a Myc- and Wnt-related developmental program. PLoS Genet 4: e10. 18208333 10.1371/journal.pgen.0040010 PMC2211538

[29] Smith JA, Wilson L, Azarenko O, Zhu X, Lewis BM, et al (2010) Eribulin binds at microtubule ends to a single site on tubulin to suppress dynamic instability. Biochemistry 49: 1331–1337. 20030375 10.1021/bi901810u PMC2846717

[30] Dong C, Li Z, Alvarez R Jr, Feng XH, Goldschmidt-Clermont PJ (2000) Microtubule binding to Smads may regulate TGF beta activity. Mol Cell 5: 27–34. 10678166 10.1016/s1097-2765(00)80400-1

[31] Cortes J, O’Shaughnessy J, Loesch D, Blum JL, Vahdat LT, et al (2011) Eribulin monotherapy versus treatment of physician’s choice in patients with metastatic breast cancer (EMBRACE): a phase 3 open-label randomised study. Lancet 377: 914–923. 21376385 10.1016/S0140-6736(11)60070-6

[32] Nozawa S, Tsukazaki K, Sakayori M, Jeng CH, Iizuka R (1988) Establishment of a human ovarian clear cell carcinoma cell line (RMG-I) and its single cell cloning–with special reference to the stem cell of the tumor. Hum Cell 1: 426–435. 3154025

[33] Yanagibashi T, Gorai I, Nakazawa T, Miyagi E, Hirahara F, et al (1997) Complexity of expression of the intermediate filaments of six new human ovarian carcinoma cell lines: new expression of cytokeratin 20. Br J Cancer 76: 829–835. 9328139 10.1038/bjc.1997.471 PMC2228076

[34] Gorai I, Nakazawa T, Miyagi E, Hirahara F, Nagashima Y, et al (1995) Establishment and characterization of two human ovarian clear cell adenocarcinoma lines from metastatic lesions with different properties. Gynecol Oncol 57: 33–46. 7535723 10.1006/gyno.1995.1097

[35] Hamilton TC, Young RC, McKoy WM, Grotzinger KR, Green JA, et al (1983) Characterization of a human ovarian carcinoma cell line (NIH:OVCAR-3) with androgen and estrogen receptors. Cancer Res 43: 5379–5389. 6604576

[36] Hamilton TC, Young RC, Ozols RF (1984) Experimental model systems of ovarian cancer: applications to the design and evaluation of new treatment approaches. Semin Oncol 11: 285–298. 6385258

[37] Provencher DM, Lounis H, Champoux L, Tetrault M, Manderson EN, et al (2000) Characterization of four novel epithelial ovarian cancer cell lines. In Vitro Cell Dev Biol Anim 36: 357–361. 10949993 10.1290/1071-2690(2000)036<0357:COFNEO>2.0.CO;2

[38] Lau DH, Lewis AD, Ehsan MN, Sikic BI (1991) Multifactorial mechanisms associated with broad cross-resistance of ovarian carcinoma cells selected by cyanomorpholino doxorubicin. Cancer Res 51: 5181–5187. 1717140

[39] Kim NW, Piatyszek MA, Prowse KR, Harley CB, West MD, et al (1994) Specific association of human telomerase activity with immortal cells and cancer. Science 266: 2011–2015. 7605428 10.1126/science.7605428

