# Supplementary figures and images for: Eribulin Mesylate Targets Human Telomerase Reverse Transcriptase in Ovarian Cancer Cells
Source: PLoS One. 2014 Nov 6;9(11):e112438. doi: 10.1371/journal.pone.0112438 (PMC4223061; doi:10.1371/journal.pone.0112438)

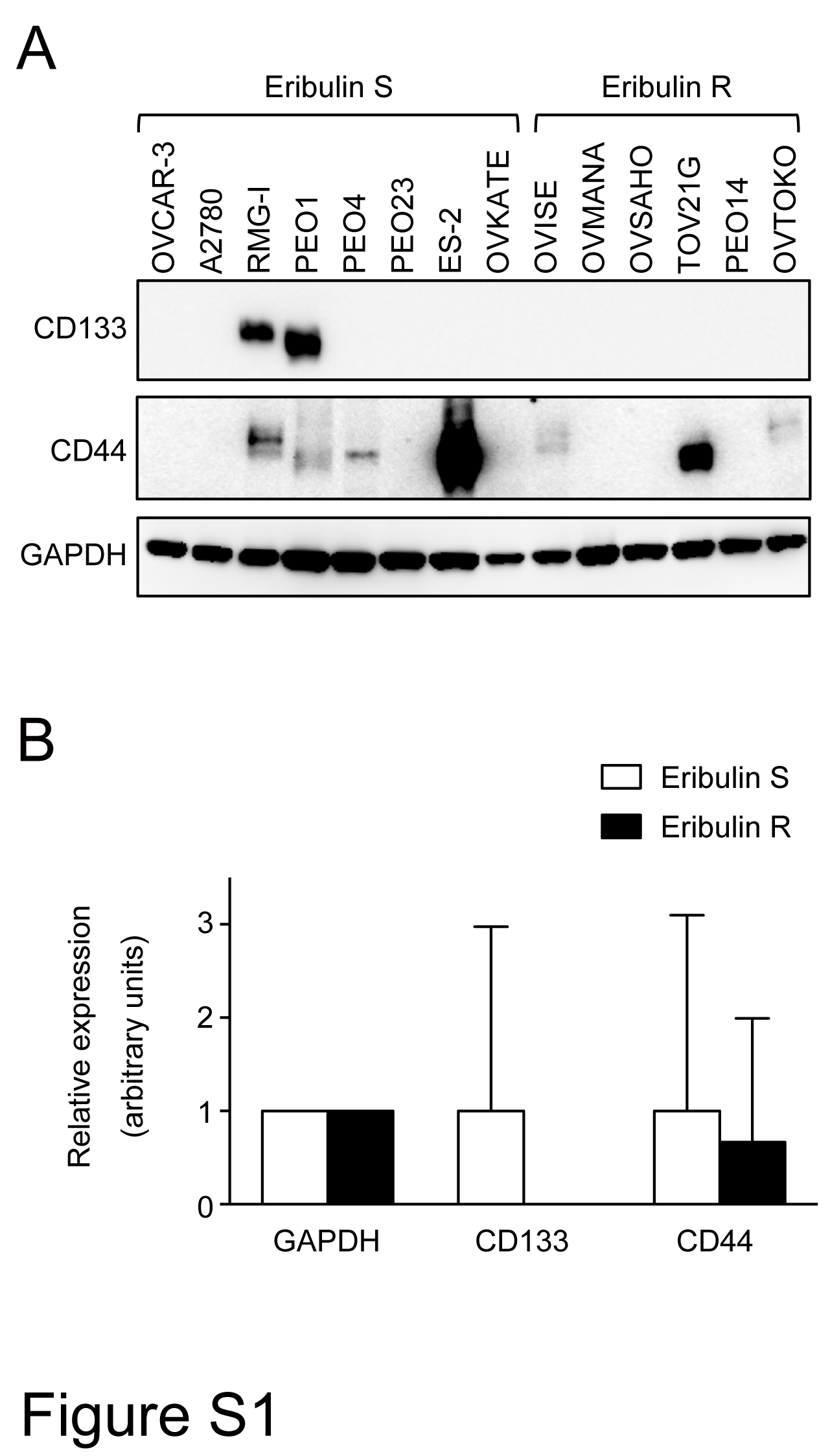

Supplement: Figure S1 — CD133 and CD44 expression in Eribulin S and Eribulin R ovarian cancer cells. (A) The level of CD133 and CD44 protein expression was detected by immunoblotting. Since the data was obtained in the same experiment as Figure 2 panel D, GAPDH gel was identical with Figure 2 panel D. (B) Signals in (A) were quantified with ImageJ software and normalized to GAPDH signal. The mean values of relative expression level ± SD are indicated. (TIF) [file pone.0112438.s001.tif]

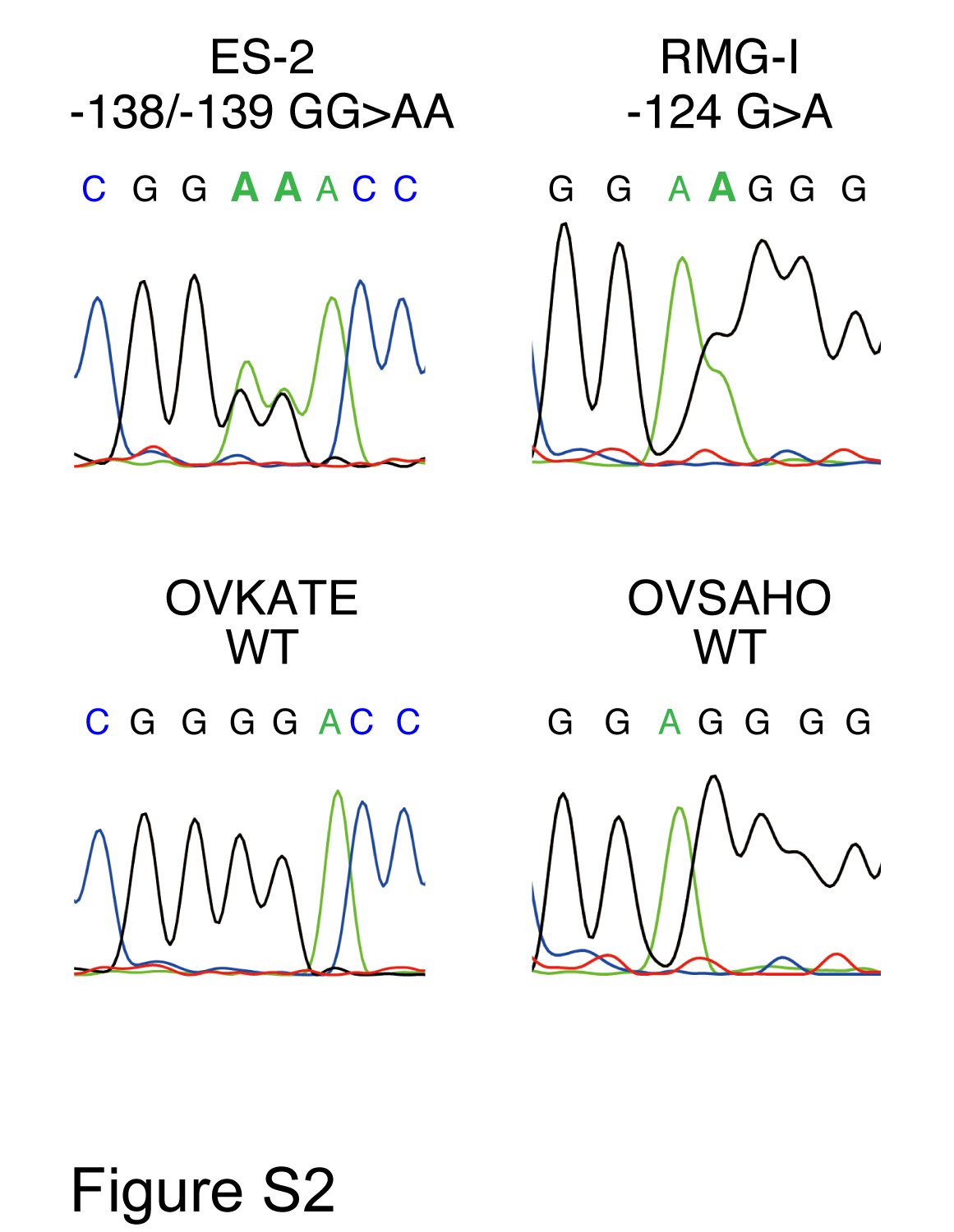

Supplement: Figure S2 — ES-2 and RMG-I cells possess hTERT promoter mutations. The hTERT promoter was sequenced in each cell line. ES-2 cells harbor a -138/-139 GG>AA mutation as described previously [27], and RMG-I cells harbor a -124 G>A mutation. The wild-type sequences of the corresponding regions from OVKATE and OVSAHO cells are shown as controls. (TIF) [file pone.0112438.s002.tif]
