# Supplementary material for: Eribulin Mesylate Targets Human Telomerase Reverse Transcriptase in Ovarian Cancer Cells
Source: PLoS One. 2014 Nov 6;9(11):e112438. doi: 10.1371/journal.pone.0112438 (PMC4223061; doi:10.1371/journal.pone.0112438)
Supplement: Table S1 — Ovarian cancer cell lines used in this study. (DOCX) [file pone.0112438.s003.docx]

**Table S1. Ovarian cancer cell lines used in this study**

| Cell line | Histological type | hTERT promoter (wild type or mutant) | Platinum-based prior treatment | References |
| --- | --- | --- | --- | --- |
| A2780 | adenoca | wild type (this study) | no | [[36](#_ENREF_36)] |
| OVCAR-3 | adenoca, P/D | wild type (this study) | yes | [[35](#_ENREF_35)] |
| PEO1 | SAC | wild type (this study) | yes | [[17](#_ENREF_17)] |
| PEO4 | SAC | wild type (this study) | yes | [[17](#_ENREF_17)] |
| PEO14 | SAC | wild type (this study) | no | [[17](#_ENREF_17)] |
| PEO23 | SAC | wild type (this study) | yes | [[17](#_ENREF_17)] |
| OVSAHO | SAC | wild type (this study) | yes | [[33](#_ENREF_33)] |
| OVKATE | SAC | wild type (this study) | yes | [[33](#_ENREF_33)] |
| OVISE | CCC | wild type ([[27](#_ENREF_27)] and this study) | yes | [[34](#_ENREF_34)] |
| OVTOKO | CCC | wild type ([[27](#_ENREF_27)] and this study) | yes | [[34](#_ENREF_34)] |
| RMG-I | CCC | -124G>A (this study) | no | [[32](#_ENREF_32)] |
| ES-2 | CCC | -138/-139GG>AA ([[27](#_ENREF_27)] and this study) |  | [[38](#_ENREF_38)] |
| TOV21G | CCC | wild type ([[27](#_ENREF_27)] and this study) |  | [[37](#_ENREF_37)] |
| OVMANA | CCC | wild type (this study) | yes | [[33](#_ENREF_33)] |

Abbreviations: adenoca, adenocarcinoma; P/D, poorly differentiated; SAC, serous adenocarcinoma; CCC, clear cell carcinoma.
